# Case Report: Late coronary complication of Kawasaki disease

**DOI:** 10.3389/fped.2025.1527714

**Published:** 2025-03-04

**Authors:** Inès Bucher, Stéphane Cook, Amel Brahim Mathiron

**Affiliations:** Department of Cardiology, University and Hospital Fribourg, Fribourg, Switzerland

**Keywords:** case report, Kawasaki disease, coronary arteries, aneurysm, myocardial ischemia

## Abstract

Kawasaki disease, a common cause of acquired heart disease in children, can lead to long-term cardiovascular complications such as coronary aneurysms and stenosis. This case describes a 17-year-old male with a past medical history of Kawasaki disease at ages 3 and 8, treated with IV immunoglobulin leading to a moderate right coronary aneurysm, who presented for a cardiac check-up. He reports only a slight dyspnoea on exertion and atypical chest pain. Despite normal findings on routine ECG and exercise tests, advanced imaging revealed significant coronary stenosis requiring angioplasty. This highlights the limitations of standard diagnostic modalities and underscores the importance of multimodal imaging and tailored management strategies in such patients.

## Introduction

Kawasaki disease was first identified in Japan in 1967, and since then, cases have been documented in more than 60 countries worldwide. The incidence varies significantly across nations, with rates ranging from 8.4 to 239.6 per 100,000 children under the age of 5 (1). Kawasaki disease is more common among certain ethnic groups, particularly in children of Asian descent. This may be explained by genetic and environmental factors. However, disparities in diagnosis and access to care persist, highlighting the importance of tailored screening strategies and transition programs for affected patients outside of high-awareness countries.

If left untreated, up to 25% of children with Kawasaki disease may develop coronary artery aneurysms, which increases their risk of cardiovascular disease (1).

Various hypotheses have been suggested to explain the long-term pathological mechanisms that may lead to cardiac complications in later life. One prevailing idea suggests subacute or chronic inflammation of the vessels, which can trigger luminal myofibroblastic proliferation, eventually causing a gradual stenosis of the coronary lumen (2). Acute coronary syndromes, including also sudden cardiac death, may occur in 1%–48% of cases, depending on the severity of secondary coronary lesions (3).

This issue is increasingly significant as the incidence of Kawasaki disease continues to rise. Furthermore, as children affected by the disease grow into adulthood, the long-term cardiac consequences are gradually beginning to manifest.

This case underscores the importance of vigilance in patients transitioning from paediatric to adult care, especially those with persistent coronary artery aneurysms. It illustrates the need for regular follow-up and advanced imaging to detect late-onset complications.

## Case description

A -17-year-old adolescent, with a history of Kawasaki disease at ages 3 and 8 presented for a cardiac check-up. During his first episode, he was treated with intravenous immunoglobulin (IVIG) and corticosteroids for persistent fever. During the second episode, 5 years later, he received IVIG, corticosteroids, and infliximab for persistent fever and clinical signs of Kawasaki disease. A moderate right coronary artery aneurysm developed (*Z*-score 6.08 according to the Paediatric Heart Network, absolute dimension of 5.2 mm by echocardiography and 5.6 mm by coronary CT at initial stage). As his aneurysms did not exceed 8 mm or a *Z*-score of 10, anticoagulation was not indicated (4). Long term treatment included low-dose aspirin. The remote assessment did not find any evidence for another vasculitis.

The patient was recently seen during health care transition, he was asymptomatic except for mild dyspnoea (NYHA I), and atypical chest pain, described as a brief pinching sensation without radiation or exertional correlation. With a weight of 67 kg and a height of 183 cm, his BMI is 20, which corresponds to a healthy weight range. He is athletic and plays basketball for a pre-professional team and has no family history of cardiovascular disease and is a non-smoker. Clinical examination was unremarkable, and he was taking 100 mg of acetylsalicylic acid daily. Laboratory tests revealed normal glycaemia, renal function and TSH. Total cholesterol is 143 mg/dl, LDL 84 mg/dl, lipoprotein (a) was elevated at 98 mg/dl, HDL 51 mg/dl, and triglycerides 59 mg/dl.

The patient presents no clinical signs of dyslipidaemia, such as xanthelasma, gerontoxon, xanthomas, or Achilles tendon thickening, and has no family history of familial dyslipidaemia. No cardiovascular risk score is applicable given his age. Due to his medical history, electrocardiography (ECG), transthoracic echocardiography, and an exercise ECG test were conducted.

## Diagnostic assessment

The initial investigations showed at ECG a new incomplete right bundle branch block without repolarization abnormalities and no Q waves. Transthoracic echocardiography revealed a preserved ejection fraction (65%) with no abnormal contractility and moderate right coronary artery dilatation (5–6 mm). Performance on the exercise ECG test was excellent with no arrhythmias or ischemic changes, achieving 203 W with fatigue as the limiting factor.

**Figure 1:**
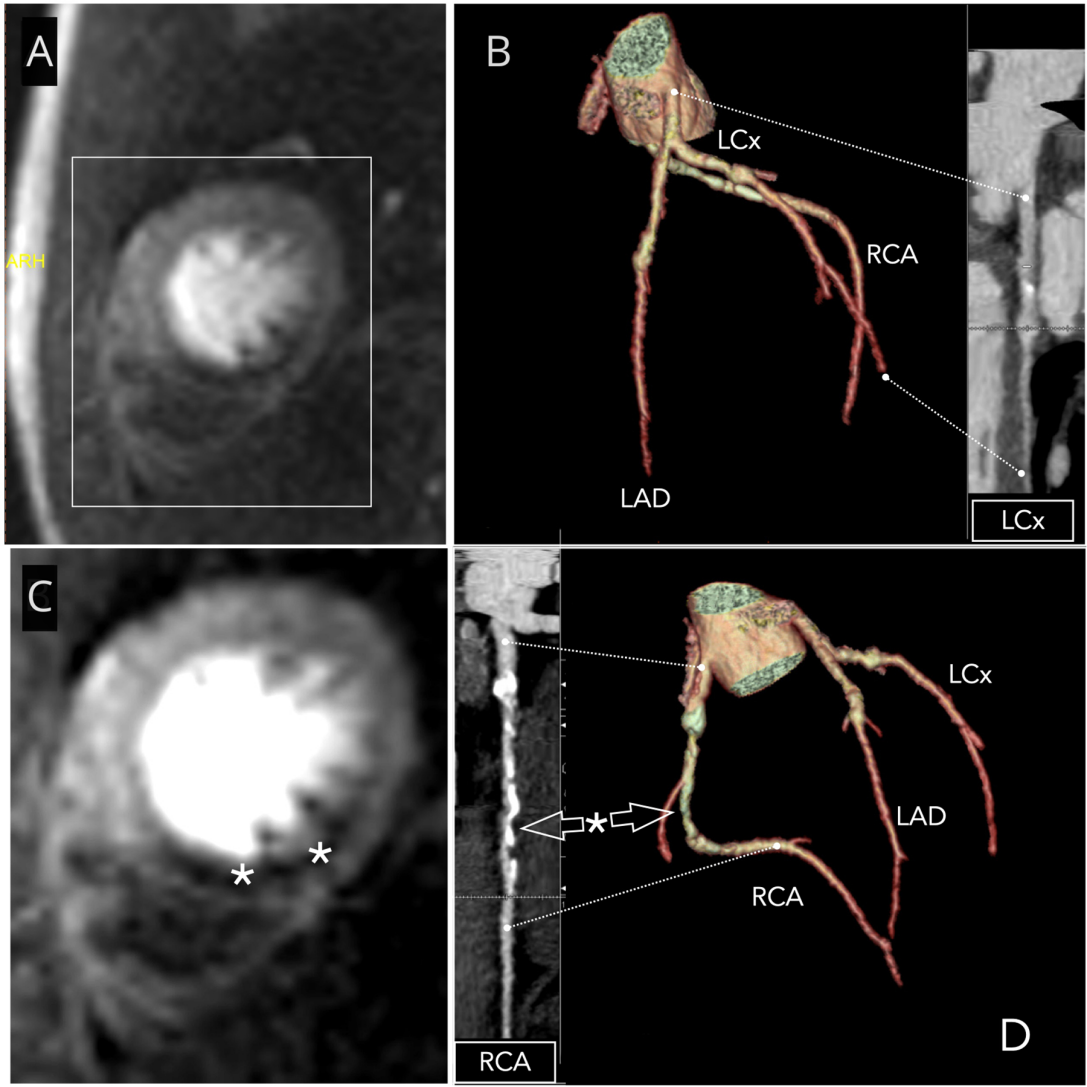
Coronary CT and stress MRI*.* **(A)** Myocardial view during stress MRI demonstrating inferior hypoperfusion (dark area) and 3D coronary CT reconstruction in LAO/cranial view **(B)**, with the longitudinal reconstruction of the circumflex branch on the right. Presence of a coronary aneurysm on the LCx branch. **(C)** Highlighted stress MRI image with asterisks identifying the region of inferior hypoperfusion and 3D coronary CT reconstruction in the anteroposterior view with longitudinal reconstruction of the right coronary artery **(D)**. Presence of a coronary aneurysm in the proximal third, numerous calcifications, and a significant stenosis (asterisk) in the mid-third. LAD, left anterior descending artery; LCx, left circumflex branch; RCA, right coronary artery.

**Figure 2:**
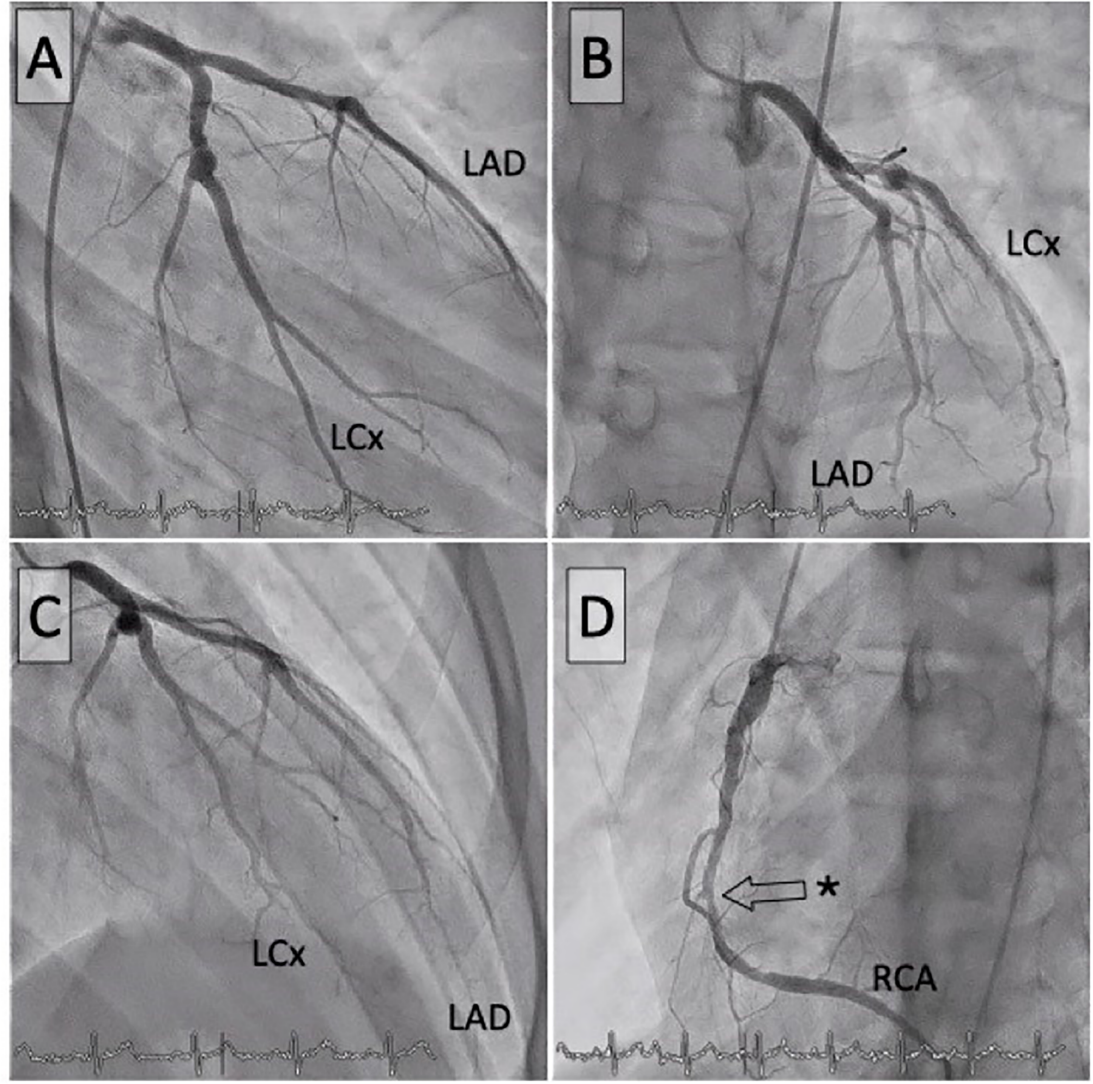
Coronary angiograms. Left coronary artery in anteroposterior view **(A)**, LAO/cranial **(B)**, RAO/cranial **(C)**, and right coronary artery **(D)**. The asterisk indicates the critical stenosis of the right coronary artery. LAD, left anterior descending artery; LCx, left circumflex branch; RCA, right coronary artery.

**Figure 3:**
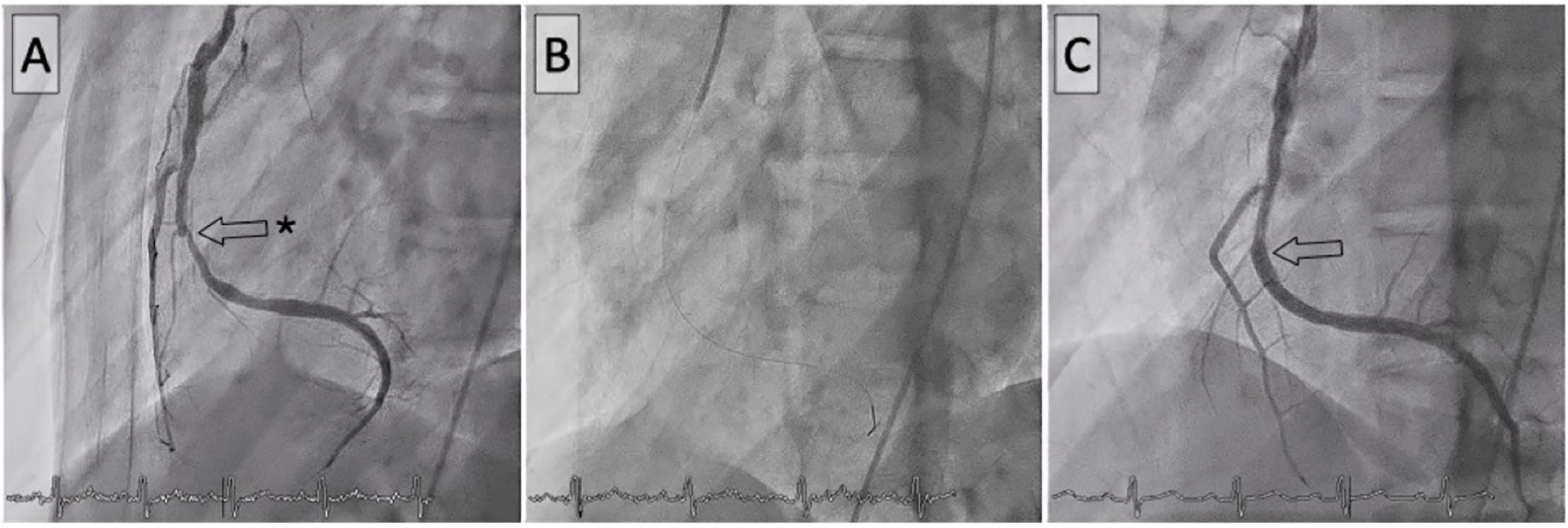
Percutaneous coronary intervention of the right coronary artery. Before **(A)**, during **(B)**, and after **(C)** stent implantation.

Given the patient's history and subtle findings on routine investigations, advanced imaging was pursued to comprehensively evaluate coronary status. Coronary CT scan revealed a high Agatston calcium score of 677, mainly affecting the right coronary, indicative of post-inflammatory sequelae, coronary narrowing, non-significant stenosis, and a medium-sized right coronary aneurysm measuring <8 mm (Figure 1). This led to the scheduling of a cardiac stress MRI with the adenosine-receptor agonist regadenoson. The MRI revealed subendocardial ischaemia in the basal inferior, septal, midventricular inferior, and particularly the apical inferior and septal regions, all showing viability and no fibrosis (Figure 1). A coronary angiogram was subsequently performed, revealing with quantitative coronary analysis a coronary artery aneurysm in the left circumflex artery (5.8 mm) and left anterior descending artery coronary (proximal LAD 6.2 mm) without significant stenosis but a severe (90%) stenosis in the mid-right coronary artery (Figure 2) without coronary artery aneurysm. The fractional flow reserve measurement indicated haemodynamic significance, with a value of 0.37. Percutaneous coronary intervention (PCI) with a third-generation drug-eluting stent was performed, with excellent immediate outcomes (Figure 3).

The patient shows no symptoms or signs of myocardial ischaemia. A follow-up stress MRI one month later showed no residual ischemia or fibrosis. The patient continues to take 100 mg of acetylsalicylic acid daily, along with 75 mg of clopidogrel. To slow disease progression, dyslipidaemia is being managed with PCSK9 inhibitors to address elevated lipoprotein(a), which is associated with an increased risk of major cardiovascular events (5).

The patient was advised on lifestyle modifications, particularly endurance exercises, and continues to receive close cardiology follow-up.

## Discussion

Kawasaki disease is an acute vasculitis of medium-sized vessels, primarily affecting children under 5. If untreated, up to 25% of patients may develop coronary artery aneurysm (CAAs), the most common cause of acquired heart disease in this population (1). Chronic inflammation and luminal myofibroblastic proliferation can lead to stenosis, calcification and ischemic events years later (2). Guidelines recommend long-term management once the acute phase has passed to prevent thrombosis and myocardial ischaemia. According to the latest guidelines from the American Heart Association (3, 4), the initial severity of coronary luminal abnormalities and their extent determine the risk of myocardial ischaemia and the need for regular follow-up. Although most coronary aneurysms are proximal (in the proximal or mid-segments of the coronary arteries), a significant fraction affects the distal segments and is often underdiagnosed with echocardiography alone.

Patients with persistent aneurysms require regular physical monitoring including echocardiography, and electrocardiography, with the frequency of these assessments depending on the severity of the lesions. Additionally, other regular cardiology tests, such as stress MRI (6) or stress echocardiography, are recommended for patients with persistent aneurysms during follow-up and in the event of any symptomatology, including atypical symptoms. Cardiovascular magnetic resonance (CMR), combined with coronary cardiovascular magnetic resonance angiography (CCMRA) if available, might offer a precise evaluation of coronary artery lumen, vessel walls, cardiac function, fibrosis, myocardial perfusion and myocardial inflammation (6, 7). While echocardiography remains a simple and easily accessible examination, CMR warrants a more accurate detection of aneurysms not identifiable on echocardiography (6). CMR may be used as a noninvasive and radiation-free imaging method for surveillance during long term follow-up (6).

Long–term management includes low-dose acetylsalicylic acid to reduce thrombotic risk. Our patient received only aspirin during long-term follow-up. In the recent statement from the American Heart Association (3, 4), both during the acute phase and long-term management, dual antiplatelet therapy may also be considered in patients at risk level 4.1 (coronary *Z* score ≥5 to <10, <8 mm and no regression as in our patient's case). Furthermore, the initiation of statin should be considered along with recurrent assessment for inducible ischemia every 2–5 years depending on the evolution and size of the aneurysm (3, 4). However, anticoagulation is only indicated if the *Z*-score is greater than 10 or if the aneurysm's absolute dimension is ≥8 mm (4). Nonetheless, the benefit-risk balance of dual antiplatelet therapy must be regularly re-evaluated due to an increased risk of bleeding in young patients during physical and sporting activities. We need further studies for the appropriate anti-inflammatory therapy to reduce the risk of coronary arteries aneurysm and late vascular complications in acute and late illness. Additionally randomized trials are necessary to assess the efficacy of dual antiplatelet therapy in patients with coronary aneurysm with a *Z*-score of ≥5. Therapies should be tailored according to the size and location of the aneurysms, drug tolerance and lifestyle.

While statins are frequently used for atheroprotection or in patients with history of Kawasaki disease with persistent aneurysms at high risk, in this case, inclisiran was considered due to its advantage of promoting good compliance with a dosing schedule of one injection every 6 months, but above all for its dual effect on both LDL and lipoprotein(a) levels. Indeed, statins could be considered as a treatment option, but they have been shown to increase lipoprotein(a) levels (8), which has been implicated as an independent risk factor for major adverse cardiovascular events (MACE) (5). Promising clinical trials are exploring targeted therapies for lipoprotein(a), such as antisense oligonucleotides (pelacarsen) and RNA interference inhibitors. These agents by specifically reducing lipoprotein(a), could ultimately complement or replace current approaches to prevent major cardiovascular events.

While data from international Kawasaki disease registry in 2020 (9) suggest that severe complications are more likely in *Z*-scores >10, this case highlights the importance of vigilance even in moderate aneurysm.

In conclusion, maintaining regular cardiac follow-up and treatment is essential for patients with a history of Kawasaki disease and persistent medium or severe coronary aneurysms. Normal results from exercise ECG tests, standard ECGs, and echocardiography should not unduly reassure clinicians and do not rule out myocardial ischaemia. The persistence of an aneurysm or myocardial ischemia should be evaluated regularly using more sensitive and multimodal imaging such as stress echocardiography, MRI or CT scan. Earlier evaluation is justified in patients with a history of Kawasaki disease in presence of typical or atypical symptoms. This vigilance enables early intervention and reduces the risk of severe late cardiovascular complications. Early identification and management of cardiovascular risk factors should be prioritised. Treatment should be individualized, balancing thrombosis prevention with bleeding risks, and addressing cardiovascular risk factor such as lipoprotein(a).

## Data Availability

The original contributions presented in the study are included in the article/Supplementary Material, further inquiries can be directed to the corresponding author.

## References

[1] Tutic E Evey K. Kawasaki disease: recent diagnostics, treatment, and predictors for treatment failure and coronary artery involvement. Curr Emerg Hosp Med Rep. (2023) 11(4):158–64. 10.1007/s40138-023-00273-4

[2] Manlhiot C Niedra E McCrindle BW. Long-term management of Kawasaki disease: implications for the adult patient. Pediatr Neonatol. (2013) 54(1):12–21. 10.1016/j.pedneo.2012.12.013 23445738

[3] McCrindle BW Rowley AH Newburger JW Burns JC Bolger AF Gewitz M Diagnosis, treatment, and long-term management of Kawasaki disease: a scientific statement for health professionals from the American Heart Association. Circulation. (2017) 135(17):e927–99. 10.1161/CIR.0000000000000484 28356445

[4] Jone PN Tremoulet A Choueiter N Dominguez SR Harahsheh AS Mitani Y Update on diagnosis and management of Kawasaki disease: a scientific statement from the American Heart Association. Circulation. (2024) 150(23):e481–500. 10.1161/CIR.0000000000001295 39534969

[5] Reyes-Soffer G Ginsberg HN Berglund L Duell PB Heffron SP Kamstrup PR Lipoprotein(a): a genetically determined, causal, and prevalent risk factor for atherosclerotic cardiovascular disease: a scientific statement from the American Heart Association. Arterioscler Thromb Vasc Biol. (2022) 42(1):e48–60. 10.1161/ATV.0000000000000147 34647487 PMC9989949

[6] Tacke CE Kuipers IM Groenink M Spijkerboer AM Kuijpers TW. Cardiac magnetic resonance imaging for noninvasive assessment of cardiovascular disease during the follow-up of patients with Kawasaki disease. Circ Cardiovasc Imaging. (2011) 4(6):712–20. 10.1161/CIRCIMAGING.111.965996 21921132

[7] Dorfman AL Geva T Samyn MM Greil G Krishnamurthy R Messroghli D SCMR expert consensus statement for cardiovascular magnetic resonance of acquired and non-structural pediatric heart disease. J Cardiovasc Magn Reson. (2022) 24(1):44. 10.1186/s12968-022-00873-1 35864534 PMC9302232

[8] Tsimikas S Gordts PLSM Nora C Yeang C Witztum JL. Statin therapy increases lipoprotein(a) levels. Eur Heart J. (2020) 41(24):2275–84. 10.1093/eurheartj/ehz310 31111151

[9] McCrindle BW Manlhiot C Newburger JW Harahsheh AS Giglia TM Dallaire F Medium-term complications associated with coronary artery aneurysms after Kawasaki disease: a study from the international Kawasaki disease registry. J Am Heart Assoc. (2020) 9(15):e016440. 10.1161/JAHA.119.016440 32750313 PMC7792232

